# Supplementary material for: Causal Relationship between Aging and Anorexia Nervosa: A White-Matter-Microstructure-Mediated Mendelian Randomization Analysis
Source: Biomedicines. 2024 Aug 16;12(8):1874. doi: 10.3390/biomedicines12081874 (PMC11351342; doi:10.3390/biomedicines12081874)
Supplement: Supplementary file 1 [file biomedicines-12-01874-s001.zip › Supplementary Figure/Figure S8/Leaveoneout plot of MR analysis for TL to ubm-b-1666.pdf]

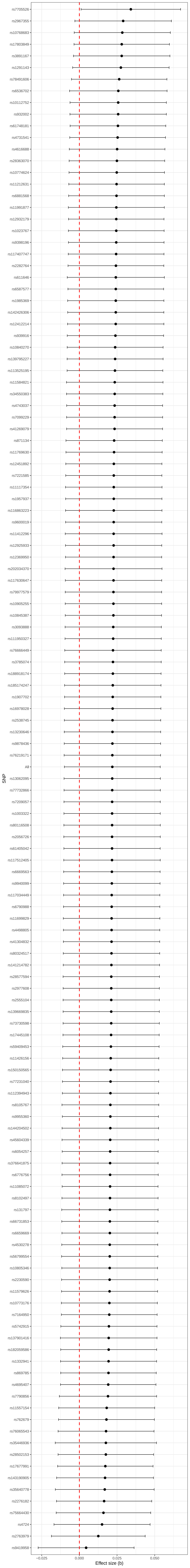
rs7705526
rs2967355
rs10768683
rs17803849
rs3891167
rs1291143
rs78491606
rs6536702
rs10112752
rs932002
rs61748181
rs4731541
rs4616688
rs28363070
rs10774624
rs11212631
rs6881568
rs11991877
rs12932179
rs1023767
rs9398196
rs117407747
rs2282764
rs611646
rs6587577
rs1985369
rs142426306
rs12412214
rs939916
rs10840270
rs139795227
rs113525195
rs11584821
rs34550383
rs4743037
rs7099229
rs41269079
rs871134
rs11769630
rs12451892
rs7221585
rs11117354
rs1957937
rs116863223
rs9600019
rs11412296
rs12925933
rs12369950
rs202034370
rs117630647
rs79977579
rs10905255
rs10845387
rs3093888
rs111950327
rs76666449
rs3785074
rs188918174
rs185174247
rs1907702
rs16978028
rs2538745
rs13230646
rs9878436
rs76219171
All
rs13062095
rs77732866
rs7209057
rs1003322
rs80116508
rs2056726
rs61405042
rs117512405
rs6669563
rs9940099
rs117034449
rs6790988
rs11699829
rs4498805
rs41304832
rs80324517
rs141214782
rs28577594
rs2977608
rs2555104
rs139669835
rs73730598
rs17445108
rs59409453
rs11426156
rs150150565
rs77231040
rs112394943
rs8105767
rs9955360
rs144204502
rs45604339
rs6054257
rs376641875
rs6776756
rs11085072
rs8102497
rs131797
rs66731853
rs6659669
rs4530278
rs56799554
rs10805346
rs2230590
rs11579626
rs10773176
rs7164950
rs5742915
rs137901416
rs182059586
rs1332941
rs869785
rs4695407
rs7790856
rs11557154
rs762679
rs76065543
rs35446936
rs28502153
rs17677991
rs143190905
rs35640778
rs2276182
rs75664430
rs4724
rs2763979
rs9419958
SNP
−0.025
0.000
0.025
0.050
Effect size (b)
